# Inhibition of ATM-directed antiviral responses by HIV-1 Vif

**DOI:** 10.1371/journal.ppat.1011634

**Published:** 2023-09-05

**Authors:** Hoi Tong Wong, Adeline M. Luperchio, Sean Riley, Daniel J. Salamango

**Affiliations:** Department of Microbiology and Immunology, Stony Brook University, Stony Brook, New York, United States of America; University of Wisconsin, UNITED STATES

## Abstract

Emerging evidence indicates that HIV-1 hijacks host DNA damage repair (DDR) pathways to facilitate multiple facets of virus replication. Canonically, HIV-1 engages proviral DDR responses through the accessory protein Vpr, which induces constitutive activation of DDR kinases ATM and ATR. However, in response to prolonged DDR signaling, ATM directly induces pro-inflammatory NF-κB signaling and activates multiple members of the TRIM family of antiviral restriction factors, several of which have been previously implicated in antagonizing retroviral and lentiviral replication. Here, we demonstrate that the HIV-1 accessory protein Vif blocks ATM-directed DNA repair processes, activation of NF-κB signaling responses, and TRIM protein phosphorylation. Vif function in ATM antagonism occurs in clinical isolates and in common HIV-1 Group M subtypes/clades circulating globally. Pharmacologic and functional studies combine to suggest that Vif blocks Vpr-directed activation of ATM but not ATR, signifying that HIV-1 utilizes discrete strategies to fine-tune DDR responses that promote virus replication while simultaneously inhibiting immune activation.

## Author summary

HIV-1 Vif facilitates virus replication by counteracting the family of APOBEC3 innate immune proteins. Recently, it has been demonstrated that Vif also induces remodeling of the host phosphoproteome by antagonizing a major phosphatase complex (PP2A); however, the virologic role of this Vif activity has yet to be established. Here, we demonstrate that Vif proteins proficient for PP2A antagonism block host DNA damage repair responses by inactivating the major DNA repair kinase ATM. In addition, functional studies using a panel of patient-derived isolates indicate that this Vif activity occurs clinically and is observed in HIV-1 subtypes/clades circulating globally. Canonically, HIV-1 induces proviral DNA repair signaling through the accessory protein Vpr, which triggers constitutive activation of DNA repair signaling responses. Prolonged DNA repair signaling is known to induce ATM-directed activation of pro-inflammatory NF-κB signaling and several members of the TRIM family of antiviral restriction factors. We demonstrate that Vif-mediated inactivation of ATM blocks the induction of these responses. These studies are important as they demonstrate that HIV-1 deploys non-overlapping strategies to fine-tune host DNA repair responses that promote virus replication while inhibiting immune activation.

## Introduction

Like all viruses, HIV-1 hijacks cellular machinery to facilitate replication and evade host antiviral defenses. To achieve this, the virus deploys several accessory proteins that enhance replication *in vitro* and *in vivo* (reviewed by [1–3]). One such protein, Vif, is required for counteracting the mutagenic potential of host antiviral APOBEC3 DNA cytosine deaminases (reviewed by [4–7]). Vif nucleates the formation of a CBF-β, ELOB/C, CUL5, and RBX2 E3-ubiquitin ligase complex to poly ubiquitinate and degrade APOBEC3s prior to virus assembly and release [8, 9]. In the absence of Vif, multiple APOBEC3 family members are capable of packaging into nascent HIV-1 particles and generating C-to-U lesions in the viral cDNA, resulting in nonsense mutations, missense mutations, and abortive integration.

Another recently ascribed Vif function is the ability to remodel the host phosphoproteome by antagonizing protein phosphatase 2A (PP2A) [10, 11]. PP2A enzymes function as heterotrimeric complexes that account for a majority of phosphatase activity in eukaryotic cells (reviewed by [12, 13]). PP2A trimers are comprised of a scaffolding protein (PPP2R1α or PPP2R1β), a phosphatase enzyme (PP2Cα), and a regulatory subunit from 1 of 3 distinct protein families (B55α-δ, B56α-ε, or PR48/59/72/130). The regulatory subunit is required for proper subcellular localization and substrate recognition by holoenzyme complexes [14, 15]. Vif antagonizes PP2A activity by targeting the family of B56α-ε regulatory proteins for proteasomal degradation through a conserved network of electrostatic interactions [16–19]. This Vif activity is observed in a variety of cell types, including primary and immortalized CD4+ T cells [10, 11, 17, 19], but the virologic function of PP2A antagonism has yet to be established.

PP2A has been implicated in regulating numerous cellular processes, including cell cycle progression, DNA damage checkpoints, and DNA repair processes, which rationalizes recent work from our group and others demonstrating an inextricable relationship between Vif-directed B56 degradation and the induction of G2/M cell cycle arrest [17–20]. In the case of DNA damage repair (DDR) responses, PP2A complexes regulate the activation of primary (ATM, ATR, DNA-PK) and secondary (CHK1 and CHK2) kinases involved in DDR signaling, as well as the recruitment of DNA repair proteins to sites of DNA damage [21–24]. This is noteworthy given that HIV-1 manipulates host DDR to facilitate genome integration and post-integration repair, minimize dead-end reverse transcription intermediates, and evade innate immune sensing [25–29]. In addition, HIV-1 accessory proteins Vpr and Vpu have been shown to directly manipulate DNA repair pathways to subvert innate immune responses [28–30].

Emerging evidence indicates that DDR responses and antiviral immunity are intimately linked [31–33]. For example, ATM engages canonical NF-κB signaling and activates several TRIM antiviral restriction factors in response to abnormal DDR signaling [34–42]. Prolonged DNA damage initiates the formation of an ATM-NEMO (NF-κB essential modulator) complex that induces the nuclear translocation of NF-κB and upregulation of IFNα, IFNλ1, IRF1, IRF7, TNFα, IL-6, and IL-8 [33, 35]. Additionally, prolonged DDR signaling also triggers ATM-directed phosphorylation and activation of TRIMs 24, 28, and 37, which have been implicated in regulating multiple facets of retrovirus and lentivirus replication [43–49].

Here, biochemical, pharmacologic, and fluorescence microscopy approaches were used to demonstrate that Vif-mediated PP2A antagonism leads to the accumulation of DNA strand breaks, likely through the inactivation of the DDR kinase ATM, in a variety of cell types. Furthermore, a peptide inhibitor known to specifically bind and inhibit B56 proteins recapitulated these observations in the absence of Vif. Additionally, functional studies using a large panel of HIV-1 group M patient-derived isolates indicate that this Vif activity occurs clinically and is observed in subtypes/clades circulating globally. Finally, we demonstrate that Vif blocks ATM-directed induction of NF-κB signaling and the phosphorylation, or degradation, of TRIM proteins previously implicated in modulating HIV-1 replication. Taken together, these results suggest that Vif-mediated inactivation of ATM counteracts inflammatory programs engaged in response to HIV-induced DDR signaling.

## Results

### Vif Inhibits the Repair of DNA Strand Breaks

**Fig 1:**
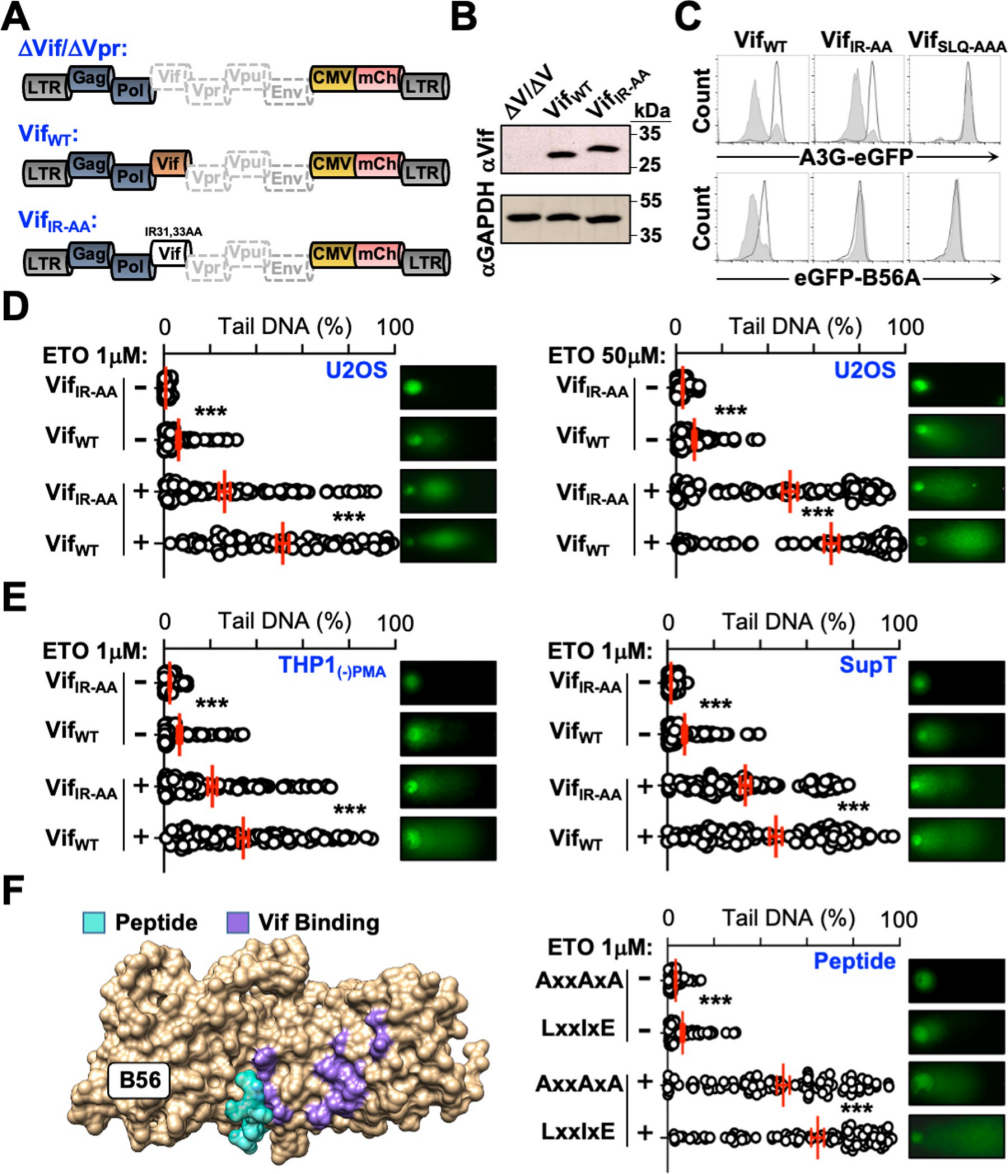
Vif expression increases DNA strand breaks. (**A**) Schematic of proviral constructs used for infection studies. (**B**) Immunoblot analysis verifying Vif expression from provirsues depicted in **1A**. (**C**) Flow cytometry histograms validating functionality of wild-type and mutant Vif derivatives tested against cells stably expressing either A3G-eGFP or eGFP-B56A (n > 20,000 cells per analysis). Open histograms (black) indicate the cell populations in the absence of Vif, whereas filled histograms (grey) indicate the presence of the indicated Vif protein. Flow cytometry experiments were repeated 3 independent times, with gating strategies, bar graph representation, and statistical analyses provided in **S1 Fig**. (**D**) Quantification and representative images for COMET assays of U2OS cells infected with the indicated viruses treated with DMSO or the indicated conentration of etoposide (ETO) (n = 50). ***, p < 0.001; ns, not significant. (**E**) Quantification and representative images for COMET assays of THP1 or SupT cells infected with the indicated viruses treated with DMSO or the indicated conentration of etoposide (ETO) (n = 50). ***, p < 0.001; ns, not significant. (**F**) Left, schematic of a B56 protein with a bound peptide inhibitor (PDB: 2JAK). Right, quantification and representative images for COMET assays of U2OS cells infected with the indicated peptide-expressing virus treated with DMSO or the indicated conentration of etoposide (ETO) (n = 50). ***, p < 0.001; ns, not significant.

Previous studies have demonstrated that PP2A complexes regulate DNA damage repair (DDR) pathways at multiple steps, and that proper resolution of each step is required for faithful DNA repair [22, 23]. Because of this, we hypothesized that expression of wild-type Vif, but not a Vif mutant defective for PP2A antagonism, would lead to inhibition of the DNA repair cascade and result in the accumulation of DNA strand breaks. To test this, we generated HIV_NL4-3_ viruses that expressed mCherry in place of Nef and were either Vif and Vpr deficient (ΔVif/ΔVpr), Vif proficient only (Vif_WT_/ΔVpr), or expressed a mutant Vif variant (Vif_IR-AA_/ΔVpr) defective for PP2A antagonism (**Figs 1A–1C and S1**). Vector functionality was confirmed by transiently transfecting HEK293T cells stably expressing eGFP-B56A or A3G-eGFP with either Vif_WT_ or mutant derivatives defective for PP2A antagonism (Vif_IR-AA_) or engagement of the E3-ubiquitin ligase complex (Vif_SLQ-AAA_ [50, 51]). To assess degradation activity, eGFP fluorescence intensity was quantified in mCherry positive cells and as expected Vif_WT_ efficiently decreased A3G and B56A eGFP fluorescence, whereas Vif_IR-AA_ could only decrease A3G-eGFP fluorescence and Vif_SLQ-AAA_ couldn’t decrease eGFP fluorescence of either substrate (**Figs 1C** and **S1**).

To determine the abundance of single-stranded/double-stranded (ss/ds) DNA breaks, alkaline COMET assays were performed on infected cells expressing either Vif_WT_, Vif_IR-AA_, or Vif_SLQ-AAA_. Proviral constructs were pseudotyped with VSV-G and overlaid onto U2OS cells at a multiplicity of infection (MOI) of 5 for 24 hours before being treated overnight with DMSO or etoposide (**Fig 1D**). As hypothesized, cells expressing Vif_WT_ but not Vif_IR-AA_ or Vif_SLQ-AAA_ exhibited a significant increase in DNA strand breaks under steady-state conditions (**Figs 1D** and **S1**). To further verify that Vif expression leads to an accumulation of DNA strand breaks cells were treated with etoposide, which induces dsDNA breaks through the inhibition of topoisomerase II. We reasoned that cells expressing Vif_IR-AA_ or Vif_SLQ-AAA_ would repair most etoposide-induced dsDNA breaks, whereas Vif_WT_ expressing cells could not. In support of this notion, treatment with low or high concentrations of etoposide exacerbated the abundance and magnitude of DNA strand breaks in Vif_WT_ expressing cells compared to Vif_IR-AA_ or Vif_SLQ-AAA_ control cells (**Figs 1D** and **S1**). Importantly, these observations were reproducible in more physiologically relevant myeloid and lymphoid THP1 and SupT cell models, respectively (**Fig 1E**).

To independently test that antagonism of PP2A-B56 containing complexes leads to an accumulation of DNA strand breaks, we used a well-characterized high-affinity peptide inhibitor. This peptide contains a conserved LxxIxE motif that directly binds the substrate recognition groove of B56 proteins, and thus effectively inactivates PP2A-B56 complexes by outcompeting interactions with cellular substrates [15, 16, 18] (**Fig 1F**). U2OS cells were infected with a lentiviral vector expressing four tandem copies of wild-type, or alanine substituted (AxxAxA), peptides fused to mCherry and subjected to COMET assays. Infected cells expressing the LxxIxE peptide exhibited significantly more DNA strand breaks in the presence or absence of etoposide compared to cells expressing the AxxAxA control peptide (**Fig 1F**). These observations support a model wherein Vif- or peptide-mediated antagonism of PP2A leads to an accumulation of DNA strand breaks.

### Vif Blocks the Formation of DNA Repair complexes

**Fig 2:**
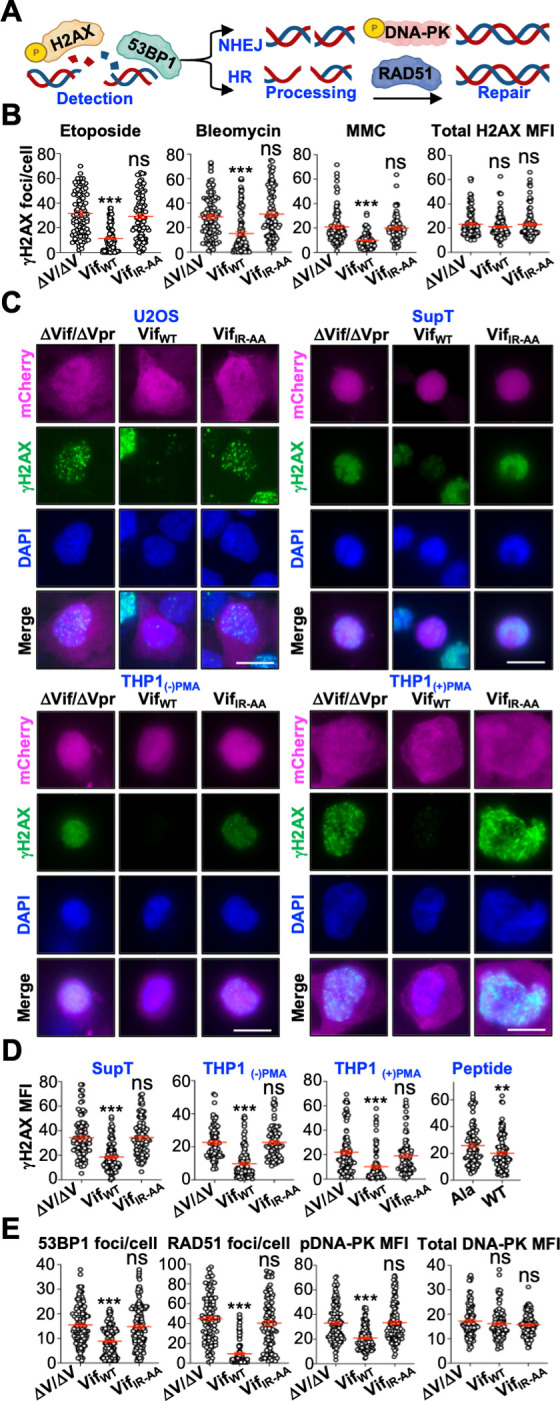
Vif inhibits the formation of DNA repair complexes. (**A**) Schematic of a simplified DNA repair signaling cascade (generated using BioRender). (**B**) Left, quantification of γH2AX (pSer 139) focus formation following etoposide, bleomycin, or mitomycin C (MMC) treatment of U2OS cells infected with the indicated viruses (n = 100). Right, mean fluorescence intensity (MFI) of total H2AX protein abundance of untreated U2OS cells infected with the indicated viruses (n = 100). For simplicity, MFI was condensed and each value is x 10^5^ arbitrary units. ***, p < 0.001; ns, not significant. Experiments were repeated 3 independent times with quantification from a single representative experiment. (**C**) Representative fluorescence miscroscopy images of the indicated cells infected with the indicated viruses. Scale bar is 10 μm. (**D**) Quantification of γH2AX mean fluorescence intensity (MFI) of the indicated cells infected with the indicated viruses following etoposide treatment (n = 100). For simplicity, MFI was condensed and each value is x 10^4^ arbitrary units. ***, p < 0.001; **, p < 0.01; ns, not significant. Experiments were repeated 3 independent times with quantification from a single representative experiment. (**E**) Left, quantification of 53BP1 foci/cell, RAD51 foci/cell, or pDNA-PK (pSer 2056) mean fluorescence intensity (MFI) of U2OS cells infected with the indicated viruses following etoposide treatment (n = 100). Right, mean fluorescence intensity (MFI) of total DNA-PK protein abundance of untreated U2OS cells infected with the indicated viruses (n = 100). For simplicity, MFI was condensed and each value is x 10^5^ arbitrary units. ***, p < 0.001; ns, not significant. Experiments were repeated 3 independent times with quantification from a single representative experiment.

To further investigate the mechanism through which Vif expression leads to an accumulation of DNA strand breaks, we utilized immunofluorescence microscopy to evaluate the activation and recruitment of DNA repair proteins to sites of DNA damage. Because etoposide induces dsDNA breaks, we focused on characterizing the recruitment of DNA repair proteins involved in non-homologous end joining (NHEJ) and homologous recombination (HR) to sites of DNA damage (**Fig 2A**). One of the earliest events in DDR initiation is the phosphorylation of histone variant H2AX at serine 139 (denoted as γH2AX), which is required for the recruitment and assembly of DNA repair complexes at sites of DNA damage. U2OS cells infected with virus expressing Vif_WT_, but not Vif_IR-AA_ or ΔVif/ΔVpr, exhibited significantly decreased γH2AX focus formation following etoposide treatment (**Fig 2B and 2C**), which was not due to decreased abundance of H2AX proteins (**Figs 2B** and **S2**). Moreover, decreased γH2AX focus formation was also observed in Vif_WT_ expressing cells treated with either bleomycin or mitomycin C, which are DNA damaging agents that induce dsDNA breaks through mechanisms distinct from that of etoposide (**Fig 2B**). Lastly, decreased γH2AX focus formation was reproducible in SupT cells, undifferentiated (monocyte-like) and differentiated (macrophage-like) THP1 cells, and in multiple cell types expressing the B56 inhibitor peptide (**Figs 2C** and **2D**, and **S2**). Phosphorylation of H2AX leads to the recruitment of the DNA damage sensor 53BP1, which dictates whether NHEJ or HR is utilized for dsDNA break repair (**Fig 2A**). In agreement with decreased γH2AX foci, we observed a significant decrease in 53BP1 focus formation and activation of downstream repair proteins involved in NHEJ (DNA-PK pSer 2056) and HR (RAD51) (**Figs 2E** and **S2**). Taken together, these findings rationalize a mechanism for the Vif-associated DNA strand breaks observed in **Figs 1** and **S1**.

### Vif-Mediated DDR Inhibition is broadly conserved

**Fig 3:**
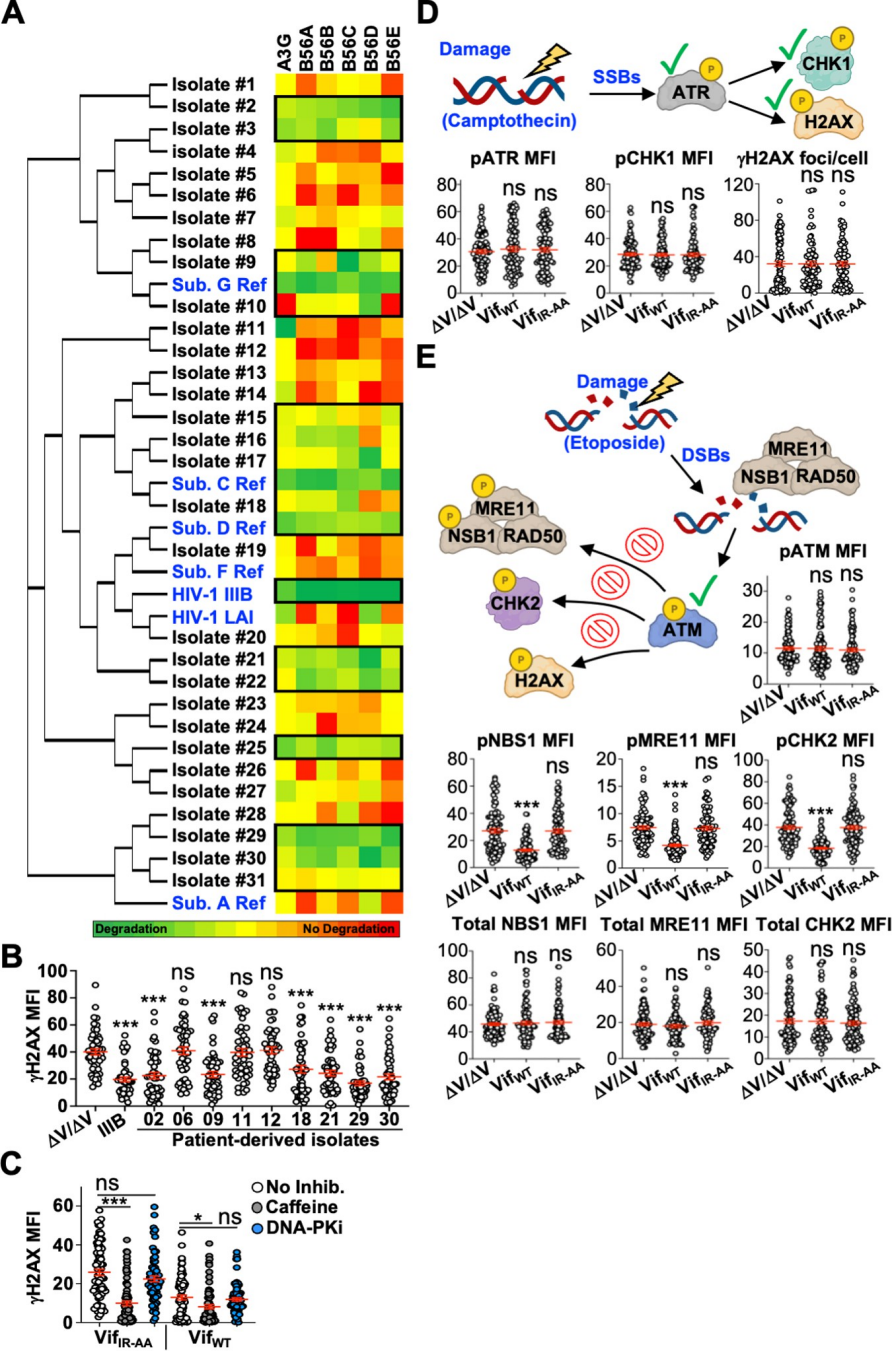
Vif inactivates the DDR kinase ATM. (**A**) Patient-derived Vif isolates and common HIV-1 consensus subtypes were examined for A3G and B56α-ε degradation activitiy. The indicated Vif construct was co-transfected with the indicated construct and eGFP mean fluorescence intensity (MFI) was quantified in mCherry-T2A-Vif expressing cells via flow cytometry (same workflow as **S1 Fig**). The mean fluorescence intensity is depicted as a heat map normalized to HIV_NL4-3_ Vif degradation activitiy with green indicating low MFI and red indicating high MFI. Black boxes highlight patients samples that demonstrates the hightest activitiy against all five B56 family members and consensus subtype sequences are depicted in blue. Flow cytometry experiments were repeated 3 independent times. (**B**) Quantification of γH2AX mean fluorescence intensity (MFI) of U2OS cells infected with the indicated mCherry-expressing or Vif-expressing viruses following etoposide treatment (n = 50). For simplicity, MFI was condensed and each value is x 10^5^ arbitrary units. ***, p < 0.001; ns, not significant. Experiments were repeated 3 independent times with quantification from a single representative experiment. (**C**) Quantification of γH2AX mean fluorescence intensity (MFI) of U2OS cells infected with the indicated viruses following etoposide treatment in the presence or absence of caffeine or a DNA-PK inhibitor (DNA-PKi) (n = 75). For simplicity, MFI was condensed and each value is x 10^5^ arbitrary units. ***, p < 0.001; **, p < 0.01; ns, not significant (additional statistics provided in **S1 Table**). Experiments were repeated 3 independent times with quantification from a single representative experiment. (**D**) Top, schematic of simplified DDR pathway focused on ATR activation (generated using BioRender). Bottom, quantification of pATR (pSer 428), pCHK1 (pSer 345) mean fluorescence intensity (MFI) or γH2AX foci/cell of U2OS cells infected with the indicated viruses following camptothecin treatment (pATR, n = 95; pCHK1, n = 100; γH2AX, n = 85). For simplicity, MFI was condensed and each value is x 10^5^ arbitrary units. ns, not significant. Experiments were repeated 3 independent times with quantification from a single representative experiment. (**E**) Top, schematic of simplified DDR pathway focused on ATM activation (generated using BioRender) with quantification of pATM (pSer 1981) mean fluorescence intensity (MFI) of U2OS cells infected with the indicated viruses following etoposide treatment (n = 100). Middle, quantification of pNBS1 (pSer 343), pMRE11 (pSer 678) or pCHK2 (pThr 68) MFI as described above (pNBS, n = 90; pMRE11, n = 90; pCHK2, n = 90). Bottom quantification of total NBS1, MRE11, and CHK2 protein abundance as described above (NBS1, n = 95; MRE11, n = 90; CHK2, n = 100). For simplicity, MFI was condensed and each value is x 10^5^ arbitrary units. ***, p < 0.001; ns, not significant. Experiments were repeated 3 independent times with quantification from a single representative experiment.

A hallmark of HIV-1 is its high degree of genetic intra- and inter-patient heterogeneity across groups, subtypes, and geographic locations. Likewise, *vif* genes exhibit high *in vivo* variability and subtype dependent amino acid substitutions; and therefore, phenotypes characterized in one strain may not adequately reflect the behavior of strains in global circulation. To address this discrepancy, we wanted to determine the prevalence of Vif-mediated DDR inhibition activity in HIV-1 group M isolates derived from patient sera collected as part of a previously characterized cohort [52]. First, we determined the frequency of PP2A antagonism activity in these isolates by assessing degradation of eGFP-tagged A3G and B56α-ε proteins. Vif isolates were cloned into a previously characterized mCherry-T2A expression construct that allows for monitoring Vif expressing cells without having an epitope directly fused to Vif [17]. Patient-derived Vif isolates were transiently co-expressed in HEK293T cells with the indicated eGFP-tagged protein, and degradation activity (loss of eGFP fluorescence) was quantified in mCherry positive cells via flow cytometry 48 hours post transfection (**Fig 3A**, color represents mean fluorescence intensity, outlines highlight Vif isolates most active against all five B56 proteins. Representative flow cytometry histograms and expression analyses are depicted in **S3 Fig**). Consensus sequences for prevalent HIV-1 group M subtypes/clades were tested in parallel for functional comparisons (highlighted in blue). Nearly all isolates tested exhibited degradation activity against A3G, and roughly half exhibited degradation activity against all five B56 family members. Importantly, many of the patient-derived isolates were closely related to reference isolates for subtypes/clades A, C, and D, which are the major strains in global circulation.

Next, several patient-derived isolates that could, or could not, efficiently degrade B56 proteins were selected for follow-up experiments to determine their effect on DDR signaling (**Fig 3B**). U2OS cells were infected with the indicated viruses for 48 hours prior to etoposide treatment and evaluation of H2AX phosphorylation. Importantly, B56 degradation activity correlated with γH2AX focus formation compared to mCherry only control cells (**Fig 3B**). These observations signify that Vif-directed inhibition of DDR signaling is present in clinical isolates and occurs in several strains circulating globally.

### Vif Inactivates the DDR Kinase ATM

H2AX can be phosphorylated by DDR kinases ATR, ATM, or DNA-PK in response to the detection of DNA strand breaks. To determine which upstream activation mechanism was inhibited by Vif, we assessed γH2AX focus formation following etoposide treatment in the presence of caffeine, which inhibits both ATM and ATR, or NU7441, which inhibits DNA-PK [53–55]. Caffeine treatment of etoposide treated Vif_IR-AA_ infected cells resulted in nearly complete ablation of γH2AX focus formation, whereas NU7441 treatment had no effect (**Fig 3C** and **S1 Table**). As observed in **Fig 2**, cells infected with Vif_WT_ exhibited significantly decreased γH2AX focus formation, which could be further reduced following treatment with caffeine but not NU7441 (**Fig 3C** and **S1 Table**). Taken together, these data are consistent with Vif blocking H2AX phosphorylation through inhibition of either ATR or ATM, but not both. If Vif were inhibiting both kinases, we would anticipate that 1) the abundance of γH2AX in Vif_WT_ expressing cells would mirror that of cells treated with caffeine alone, and 2) that caffeine treatment of Vif_WT_ expressing cells would have no impact on γH2AX levels.

Next, we wanted to determine which kinase was inhibited by Vif. First, we assessed ATR activation by treating infected cells with camptothecin or etoposide. Camptothecin inhibits topoisomerase I to induce replication stress, ssDNA breaks, and activation of ATR and ATR-directed signaling (but not the activation of ATM). As depicted in **Figs 3D** and **S4**, no differences in ATR phosphorylation (pSer 428) were observed in U2OS cells infected with ΔVif/ΔVpr, Vif_WT_, or Vif_IR-AA_ following camptothecin or etoposide treatment. Likewise, camptothecin-induced phosphorylation of downstream targets CHK1 (pSer 345) and H2AX were also unaffected in Vif_WT_ expressing cells (**Figs 3D** and **S4**), suggesting that Vif-mediated inhibition of DNA repair occurs through inactivation of ATM.

Detection of dsDNA breaks by the MRN (MRE11/RAD50/NBS1) complex leads to ATM transphosphorylation and the release of active ATM monomers from inactive homodimers [56]. Therefore, we investigated ATM phosphorylation following etoposide treatment of infected U2OS cells; and surprisingly, no differences were observed between ΔVif/ΔVpr, Vif_WT_, or Vif_IR-AA_ infected cells (**Figs 3E** and **S5**). These observations were perplexing given that COMET assays, inhibitor treatments, and DNA damage repair studies collectively indicated that Vif blocks DDR through ATM inactivation. Upon further investigation, recent biochemical, structural, and functional studies indicate that ATM transphosphorylation may not accurately reflect kinase activity *in vitro* or *in vivo* [57–61]. Therefore, to further evaluate ATM functionality, we examined the phosphorylation of additional ATM targets NBS1 (pSer 343), MRE11 (pSer 678), and CHK2 (pThr 68) following etoposide treatment. All three substrates exhibited significantly decreased phosphorylation in Vif_WT_ but not ΔVif/ΔVpr or Vif_IR-AA_ infected cells, further suggesting that Vif inhibits DDR through inactivation of ATM (**Figs 3E** and **S5**). Of note, total protein of all three targets remained unchanged under these experimental conditions (**Figs 3E** and **S5**).

### Vif Counteracts a Subset of Vpr-Directed DDR Responses

**Fig 4:**
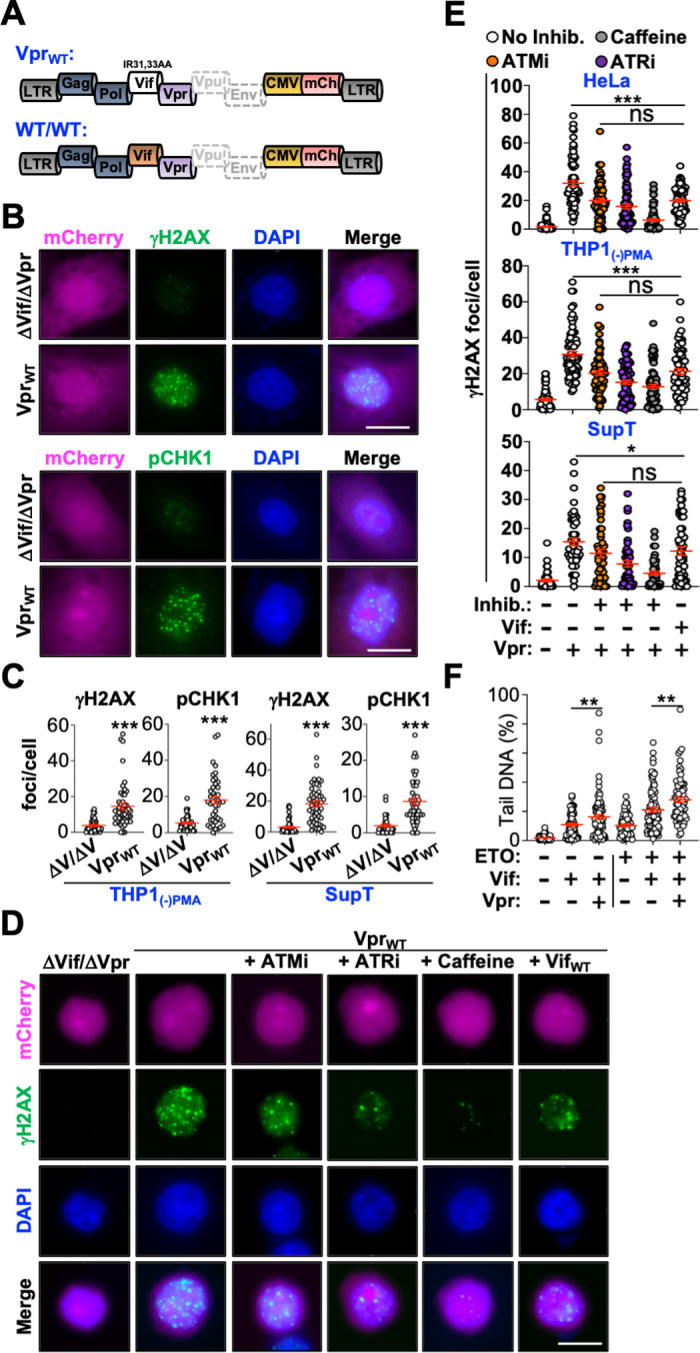
Vif counteracts Vpr-induced ATM activation. (**A**) Schematic of proviral constructs used for infection studies. (**B**) Representative fluorescence microscopy images of HeLa cells infected with the indicated viruses. Scale bar is 10 μm. (**C**) Quantification of γH2AX or pCHK1 foci/cell from the indicated cell line infected with the indicated viruses (n = 30). ***, p < 0.001. Experiments were repeated 3 independent times with quantification from a single representative experiment. (**D**) Representative fluorescence mcroscopy images of THP1 cells infected with the indicated viruses in the presence and absence of the indicated inhibitors. Scale bar is 10 μm. ATMi, ATM inhibitor; ATRi, ATR inhibitor. (**E**) Quantification of γH2AX foci/cell in the indicated cell line infected with the indicated viruses in the presence or absence of inhibitor treatment (n = 50). ***, p < 0.001; ns, not significant (additional statistics provided in **S1 Table).**ATMi, ATM inhibitor; ATRi, ATR inhibitor. Experiments were repeated 3 independent times with quantification from a single representative experiment. (**F**) Quantification for COMET assays of HeLa cells infected with the indicated viruses treated with DMSO or etoposide (n = 50). **, p < 0.01.

HIV-1 primarily engages host DDR through the accessory protein Vpr, which activates DDR signaling, inhibits repair of diverse DNA lesions, and induces DNA strand breaks [62–65]. Vpr-directed DDR activation is a highly conserved function that triggers constitutive activation of ATM, ATR, and downstream factors such as H2AX and CHK1 [64, 66–69]. To determine the impact of Vif and Vpr co-expression on DDR responses, we generated additional mCherry reporter viruses that express Vpr/Vif_IR-AA_ or co-express Vpr and Vif (**Fig 4A**). First, we established that these Vpr expressing proviruses were capable of inducing DDR responses in cell models used for Vif studies described above. Viral stocks were generated and used to infect HeLa, THP1, or SupT cells for 48 hours prior to evaluating phosphorylation of H2AX, CHK1, and CHK2. In the absence of a DNA damaging agent, significant accumulation of γH2AX, pCHK1, and pCHK2 foci were observed in Vpr/Vif_IR-AA_ expressing cells, but not in control cells infected with the ΔVif/ΔVpr virus (**Figs 4B** and **4C** and **S5**), indicating that these cell models are amenable for investigating the impact of Vif and Vpr co-expression on DDR.

Next, we wanted to determine if Vif-mediated inhibition of ATM was maintained in the presence of Vpr. For these experiments, we primarily focused on γH2AX as an experimental readout as H2AX can be phosphorylated by both ATM and ATR; and therefore, allows for assessing the contribution of each kinase to Vpr-directed DDR responses in the presence and absence of Vif. Vpr/Vif_IR-AA_ infected HeLa, THP1, or SupT cells were treated with vehicle, an ATM inhibitor (AZD1390) [70, 71], an ATR inhibitor (NU6027) [72], or caffeine, and γH2AX foci were quantified 48-hours post-infection (note: these cells were not treated with a DNA damaging agent). In the absence of an inhibitor, all three cell lines exhibited significantly increased γH2AX foci in Vpr/Vif_IR-AA_ expressing cells compared to ΔVif/ΔVpr control cells (**Fig 4D and 4E**, and **S1 Table**). Following inhibitor treatment, the abundance of γH2AX foci were significantly reduced with caffeine treated cells exhibiting the greatest degree of inhibition (∼85%), followed by ATR inhibitor treatment (∼60%), and ATM inhibitor treatment (∼30%). Importantly, co-expression of Vif and Vpr resulted in reduced γH2AX focus formation to a level comparable to that of the ATM inhibitor treatment. While these observations are not definitive, they are consistent with the idea that Vif expression inhibits Vpr-directed activation of ATM but not ATR (**Fig 4D and 4E**, and **S1 Table**). These observations were also reproducible when assessing Vpr-induced phosphorylation of CHK2 (**S5 Fig**). To further determine if Vif and Vpr block DDR responses independently, we evaluated DNA strand breaks in cells infected with ΔVif/ΔVpr, Vif only, or Vif and Vpr expressing viruses in the presence and absence of etoposide treatment. As depicted in **Fig 4F**, co-expression of Vif and Vpr resulted in significantly more DNA strand breaks compared to Vif alone in the presence or absence of etoposide, further suggesting that Vif and Vpr block DNA repair through independent mechanisms.

### Vif Inhibits ATM-Directed antiviral responses

In response to abnormal activation of DDR signaling, ATM engages diverse antiviral defense mechanisms, including the activation and nuclear translocation of NF-κB, STAT1/2, IRF1/7, increased ROS production, and upregulation of TNFα, IL-6, and IL-8 [31–37, 70]. In addition, ATM directly phosphorylates several members of the TRIM family of antiviral restriction factors, including TRIMs 24, 28, and 37, which have been previously implicated in regulating multiple facets of retrovirus and lentivirus replication [43–49]. Therefore, we hypothesized that Vif-directed inhibition of ATM blocks the activation of these antiviral programs in response to constitutive DDR signaling.

**Fig 5:**
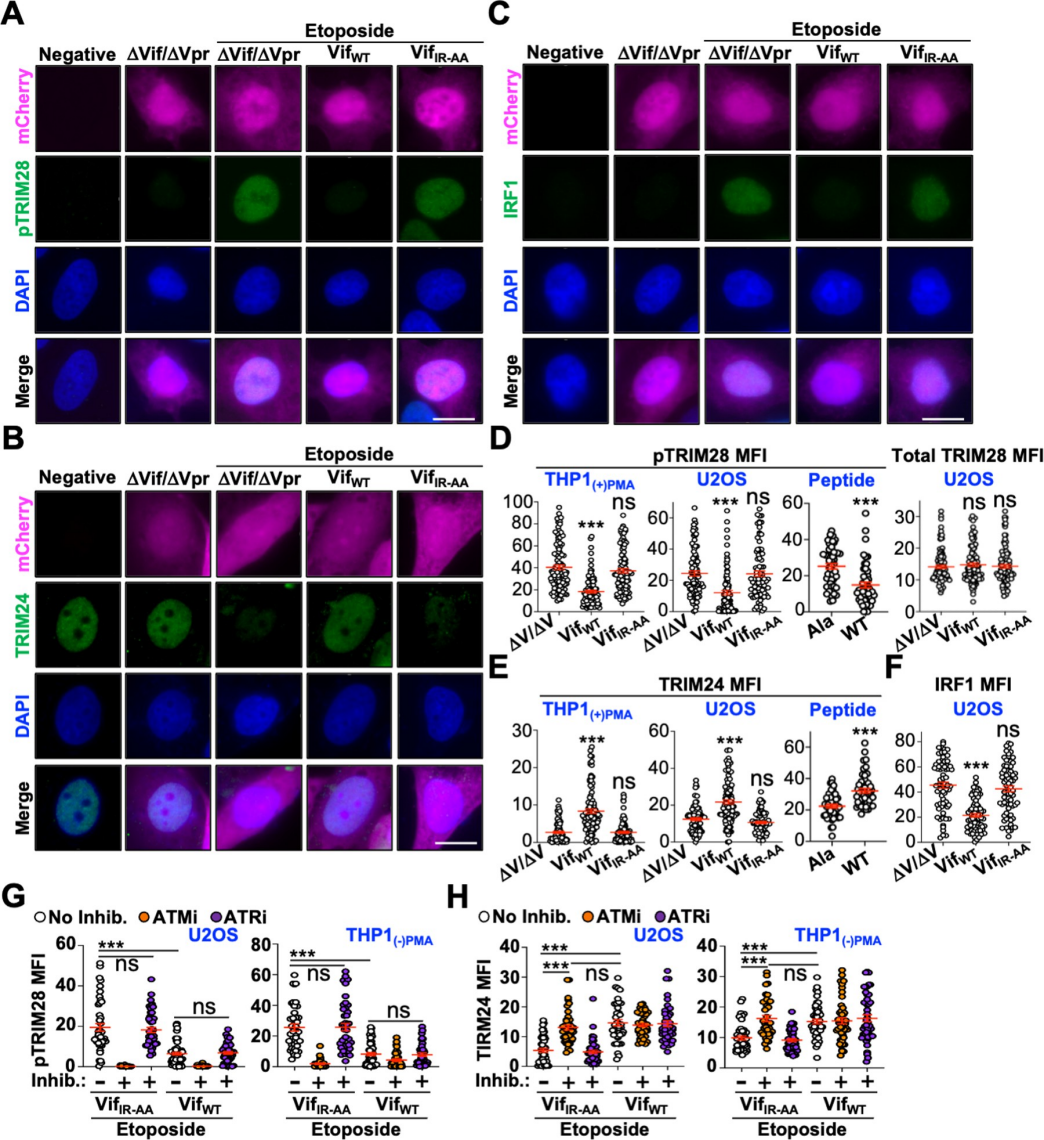
Vif inhibits ATM-directed inflammatory programs. (**A**-**C**) Representative fluorescence microscopy images of U2OS cells that are uninfected or infected with the indicated viruses with and without etoposide treatment. TRIM28 phosphorylation was assessed following a 30-minute treatment, TRIM24 depletion was following a 4 hour treatment, and IRF1 was following a 24 hour treatment. Scale bar is 10 μm. (**D**-**F**) Quantification of pTRIM28 (pSer 824), total TRIM28, total TRIM24, or total IRF1 following the experimental procedure as outlined above (n = 100). ***, p < 0.001; ns, not significant. Experiments were repeated 3 independent times with quantification from a single representative experiment. (**G** and **H**) Quantification of pTRIM28 and total TRIM24 following the experimental procedure as outlined above in the presence or absence of the indicated inhibitor (n = 50). ***, p < 0.001; ns, not significant (additional statistics provided in **S1 Table**). ATMi, ATM inhibitor; ATRi, ATR inhibitor. Experiments were repeated 3 independent times with quantification from a single representative experiment.

To test this hypothesis, we examined the activation of two TRIM proteins previously implicated in modulating HIV-1 replication. In response to DNA damage, ATM rapidly phosphorylates TRIM28 at serine 824 while it directs TRIM24 to the proteasome via phosphorylation of serine 768. Differentiated THP1 or U2OS cells were infected with ΔVif/ΔVpr, Vif_WT_/ΔVpr, or Vif_IR-AA_/ΔVpr viruses for 48 hours prior to etoposide treatment and evaluation of TRIM28 phosphorylation or TRIM24 depletion. In both cell types, expression of Vif_WT_/ΔVpr but not Vif_IR-AA_/ΔVpr resulted in significantly decreased TRIM28 phosphorylation, with no change in TRIM28 protein abundance (**Fig 5A and 5D**). Because ATM directs proteasomal degradation of TRIM24 in response to prolonged DNA damage, we anticipated that Vif_WT_/ΔVpr expression would lead to increased abundance of TRIM24 proteins following etoposide-induced depletion. As expected, significantly more TRIM24 protein was detectable in both U2OS and THP1 cells infected with Vif_WT_/ΔVpr but not with Vif_IR-AA_/ΔVpr or ΔVif/ΔVpr (**Fig 5B and 5E**). Importantly, these observations were reproducible in cells infected with the wild-type peptide inhibitor compared to the alanine peptide control, further suggesting that antagonism of PP2A-B56 complexes is driving ATM inhibition (**Fig 5D and 5E**). Lastly, we sought to determine if Vif could also inhibit ATM-induced NF-κB responses. Etoposide is known to induce the accumulation of interferon stimulated genes, interferon regulatory factors IRF1 and IRF7, and IFNα and IFNλ1 [33]. For these studies, U2OS cells were infected for 24 hours prior to a 24-hour etoposide treatment to induce NF-κB activation. As depicted in **Fig 5C and 5F**, IRF1 accumulation was detectable in etoposide treated cells expressing ΔVif/ΔVpr or Vif_IR-AA_/ΔVpr, but not in cells expressing Vif_WT_/ΔVpr, suggesting that Vif also blocks ATM-directed NF-κB activation.

To confirm that etoposide-induced activation of these inflammatory programs occurred through an ATM-dependent mechanism, TRIM28 phosphorylation and TRIM24 depletion were evaluated in the presence and absence of ATM and ATR inhibitors. U2OS and THP1 cells infected with Vif_IR-AA_/ΔVpr exhibited robust TRIM28 phosphorylation following etoposide treatment in the absence of inhibitor. However, TRIM28 phosphorylation was completely ablated in cells treated with the ATM inhibitor, while ATR inhibitor treatment had no effect (**Fig 5G** and **S1 Table**). Likewise, cells infected with Vif_WT_/ΔVpr exhibited a similar pattern of TRIM28 phosphorylation in the presence and absence of inhibitor treatments (**Fig 5G** and **S1 Table**). Furthermore, ATM inhibitor treatment reversed etoposide-induced loss of TRIM24 protein abundance in U2OS and THP1 cells expressing Vif_IR-AA_/ΔVpr, while untreated and ATR inhibitor treated cells exhibited significant depletion of TRIM24 proteins (**Fig 5H** and **S1 Table**). Vif_WT_/ΔVpr expressing cells exhibited a similar amount of TRIM24 protein accumulation under all treatment conditions (**Fig 5H** and **S1 Table**). Taken together, these observations suggest that Vif inhibits multiple ATM-directed antiviral responses.

## Discussion

Although Vif has been extensively characterized for its ability to target and degrade APOBEC3 restriction factors, the functional relevance of PP2A antagonism has yet to be established. Here, we show that Vif-mediated antagonism of PP2A inactivates the DNA repair kinase ATM, leading to the inhibition of DNA repair complexes and accumulation of DNA strand breaks. Similar DNA repair defects were recapitulated using a B56-specific peptide inhibitor and pharmacologic inhibition of ATM. Functional studies and phylogenetic analyses combined to indicate mechanistic conservation in patient-derived Vif isolates and in common HIV-1 group M subtypes/clades circulating globally. Co-expression of Vif and Vpr resulted in significantly more DNA strand breaks compared to expression of either protein alone, and revealed that Vif counteracts Vpr-induced activation of ATM but not ATR, signifying that HIV-1 utilizes non-overlapping strategies to antagonize host DNA repair. Importantly, Vif-mediated inhibition of ATM counteracted ATM-directed induction of NF-κB signaling and activation of TRIM antiviral restriction factors, defining a functional role for PP2A antagonism in HIV-1 replication.

Our findings support a model in which Vif and Vpr fine-tune DDR responses to promote virus replication while simultaneously inhibiting activation of antiviral responses. Prolonged activation of DDR triggers the formation of an ATM-NEMO complex that stimulates pro-inflammatory NF-κB signaling in addition to the activation of TRIMs 24, 28, and 37. Consistent with previous studies, we demonstrate that Vpr expression leads to the activation of ATM- and ATR-mediated signaling, and that Vif co-expression inhibits a subset of these responses. Interestingly, a recent preprint demonstrated that AAV-delivered (adeno-associated virus) Vpr altered the cellular transcriptome through an ATM-NEMO-NF-κB dependent signaling axis [73]. In addition, transcriptional upregulation required Vpr-induced DNA damage and activation of DDR signaling. Follow-up studies will be necessary to determine if co-expression of Vif and Vpr alters these transcriptional responses, as the HIV-directed experiments in this study utilized HIV_LAI_, whose Vif protein does not antagonize PP2A.

In response to abnormal DDR activation, ATM phosphorylates and activates TRIMs 24, 28, and 37, which have been implicated in regulating retroviral and lentiviral replication. In general, these TRIMs regulate integration and transcription of the viral genome and promoter, respectively. TRIM37 is also involved in the formation of the ATM-NEMO complex to facilitate NF-κB activation [42]. Interestingly, TRIMs 24 and 28 have opposing functions with respect to regulating HIV-1 promoter activity. TRIM28 has been well characterized as being a negative regulator of HIV-1 integration and transcription activity in both lymphoid and myeloid cells [44, 46]. Post-integration, TRIM28-mediated antagonism of the HIV-1 promoter drives a latency-like phenotype through inhibition of CDK9-pTEFb binding [47]. While ATM-mediated phosphorylation of TRIMs 28 and 37 activates their antiviral activity, phosphorylation of TRIM24 triggers its proteasomal degradation [39]. Under steady-state conditions, TRIM24 is recruited to the HIV-1 promoter through interactions with CDK9-pTEFb to stimulate transcriptional elongation [48, 49]. As is the case with TRIM28, TRIM24 functions to control HIV-1 promoter activity to prevent or induce viral latency.

In line with coordinated dysregulation of DNA repair, Vif and Vpr also independently induce a G2/M cell cycle arrest phenotype. While the mechanistic details of how Vpr induces G2/M arrest are debated, it is clear that it requires ATR activity, but not ATM activity, to induce arrest. For Vif, recent work from our lab and others has established that Vif-induced G2/M arrest requires PP2A antagonism, but the exact arrest mechanism has yet to be elucidated [74]. Given that Vpr-induced arrest requires DDR antagonism, and that the DNA damage response is intimately linked to cell cycle regulation, it is plausible that Vif’s ability to inactivate ATM may contribute to its G2/M arrest activity. Future dedicated studies will be required to establish if Vif-directed DDR antagonism is required for its G2/M arrest activity, or, if PP2A antagonism alone is sufficient.

Vif’s ability to induce cell cycle arrest may also provide an explanation for the lack of ATM activity observed in these studies even though transphosphorylation is intact. In addition to phosphorylation, ATM requires acetylation at lysine 3016 for kinase activity [75, 76]. In response to DNA damage, the acetyltransferase TIP60 acetylates several targets involved in DDR, including ATM and H2AX [75, 77, 78]. TIP60 activation occurs through a CDK1-dependent mechanism [79], which along with Cyclin B1, is known to be antagonized by Vif [80]. TIP60 also regulates cell cycle progression through acetylation of Aurora kinase B, which is a known PP2A target aberrantly activated in the presence of Vif [10, 11, 81]. Furthermore, c-Myc, which is also regulated by PP2A, influences CDK1 dependent G2/M cell cycle progression through histone H4, which is another acetylation target of TIP60. Lastly, TIP60 and CyclinB1 are also known interacting partners of HIV-1 Tat, raising the possibility that Vif and Tat might coordinate to dysregulate DDR responses [82, 83].

Subversion of host DNA repair mechanisms to facilitate virus replication and immune evasion has been observed extensively across diverse viral families, including ssDNA and dsDNA viruses, positive and negative stranded RNA viruses, and amongst other retroviruses [84]. These mechanisms encompass the induction of diverse DNA lesions during virus replication [*ex*., HIV-1, human T-lymphotropic virus (HTLV), influenza A, and simian virus 40,], direct manipulation of DNA repair complexes and signaling cascades [*ex*., adenovirus, Epstein-Barr virus, HIV-1, human papilloma virus, HTLV, Kaposi’s Sarcoma-associated Herpesvirus (KSHV), and rotavirus], and the suppression of DDR-directed immune responses (*ex*., HIV-1, HTLV, and KSHV). Taken together, these observations support the notion that hijacking host DNA repair responses is a broadly conserved mechanism to boost the pathogenicity of diverse viral families.

## Materials and methods

### Plasmids and cloning

Expression plasmids used in this study were either cloned into a pQCXIH retroviral expression vector, an envelope deficient NL4-3 proviral derivative that expresses CMV-driven mCherry in place of Nef, or a lentiviral CMV-driven mCherry-T2A expression cassette [17, 85, 86]. The cDNA sequences for all *PPP2R5* genes were ordered as gblocks from Integrated DNA Technologies (IDT) and cloned in-frame with an eGFP coding sequence using *Age*I and *Bsi*WI restriction sites. The pQCXIH APOBEC3G-eGFP expression vector has been described [87]. The HA-epitope tagged Vif derivates utilized for immunoblot analyses were generated through PCR amplification using a forward primer with a single copy of an HA-tag in-frame with Vif. Vif and Vpr mutants were generated by PCR amplification using Phusion high fidelity DNA polymerase (NEB, Ipswich, MA) and overlapping PCR to introduce the indicated mutations. All constructs were confirmed by restriction digestion and Sanger sequencing. Patient-derived Vif isolates were obtained from a previously characterized cohort and their sub-cloning is described in detail here [16]. Consensus sequences for common HIV-1 subtypes were based on sequences obtained from the Los Alamos database and were synthesized as gblocks from IDT.

### Cell lines and culture conditions

U2OS, HeLa, HEK293T cells (American Type Culture Collection) were maintained in DMEM medium (Gibco; cat #11-965-118) supplemented with 10% fetal bovine serum (FBS; Gibco, Gaithersburg, MD) and 0.5% penicillin-streptomycin (50 units; Gibco, Gaithersburg, MD). SupT and THP1 cells (American Type Culture Collection) were maintained in RPMI medium supplemented with 10% FBS and 0.5% penicillin-streptomycin. HEK293T cells were transfected with polyethylenimine (PEI; Fisher #NC1014320) using a ratio of 3 μL per 1 μg of DNA. To generate viruses, HEK293T cells were co-transfected with a VSV-G expression vector along with a proviral plasmid expressing the indicated Vif and Vpr variants and a CMV-driven mCherry reporter in place of Nef. Medium was collected 48 h post transfection and frozen at -80°C until used. For THP1 differentiation, cells were incubated with 50–70 ng/ml of phorbol 12-myristate 13-acetate (PMA; Sigma #P8139) for 48 h.

To generate stable PP2A eGFP-B56A and APOBEC3G-eGFP cell lines, viruses were produced from 293T cells transfected with the pQCXIH retroviral expression vectors described above, an MLV GagPol packaging vector, and a VSV-G vector. Media was harvested 48 hours post-transfection and frozen at -80°C for 4–6 hours, thawed and centrifuged at 1500 x *g*, and combined with fresh 293T cells. To generate pure cell populations, samples were treated with hygromycin B (Sigma, 200 mg/ml) 48 hours post-transduction.

### Fluorescence microscopy and immunostaining

For all immunofluorescence experiments with U2OS and HeLa cell lines, approximately 8,000 cells were seeded into an eight-well glass bottom chamber slide (Ibidi #80826) and allowed to adhere overnight. The next day, cells were infected with the indicated viruses for 48 h. Prior to staining, cells were treated with vehicle or the indicated DNA damaging agent in the presence or absence of the indicated inhibitor. Experiments assessing total protein abundance followed the same incubation and infection timeline, but samples were left untreated. Immunostaining of the indicated DNA repair markers was performed as follows. At 48 hours post infection, cells were washed with PBS and fixed in 4% paraformaldehyde (PFA) at room temperature for 10 min. Following fixation, cells were washed three times with PBS in 5-minute intervals and then permeabilized using PBS plus 0.3% Triton X-100 (PBST) for 10 minutes at room temperature. Cells were blocked using PBST supplemented with 5% BSA, 10% goat serum and 0.3 M glycine for 2 h at room temperature. After blocking, samples were incubated with primary antibodies γH2AX (1:300, Cell Signaling 9718), pCHK1 (1:50, Cell Signaling 2348), pCHK2 (1:200, Cell Signaling 2661), pATM (1:300, Cell Signaling 4526), pATR (1:300, Cell Signaling 2853), pTRIM28 (1:500, Thermo A300-767A), pMRE11 (1:50, Cell Signaling 4859), MRE11 (1:200, Cell Signaling 4847), pNBS1 (1:200, Cell Signaling 3001), NBS1 (1:200, Novus Biologicals NB100-143), TRIM24 (1:200, Proteintech 14208-1-AP) in blocking buffer overnight at 4°C. The next day, samples were washed three times with PBS in 5-minute intervals and then incubated with anti-mCherry conjugated to Alexa Fluor 594 (1:800; Invitrogen M11240) and secondary anti-rabbit-IgG conjugated to Alexa Fluor 488 (1:800, Cell Signaling 4412) in blocking buffer for 1 h at room temperature. Cells were washed three times with PBS at 5-minute intervals before staining with DAPI (nucleus) and imaging. Cells were imaged on an EVOS M500 fluorescence microscope using a 60x oil-immersion objective.

For immunofluorescence microscopy using SupT and THP1 cells, approximately 75,000–100,000 cells were seeded and co-infected with the indicated viruses in a 12-well tissue culture plate. Prior to immunostaining, cells were treated with appropriate drugs and inhibitors for indicated times. Cells were then processed for immunofluorescence as described above, with the modification that in between steps the cells were pelleted through centrifugation at 500 x g for 5-minute intervals. Prior to imaging, cells were transferred to a 96-well glass bottom imaging plate (Ibidi #89626). All experiments were repeated at least 3 independent times between two investigators. For quantification, all experimental samples contain between 50–100 independent cell nuclei.

### Comet assays

For detection of DNA strand breaks, comet assays were performed using indicated cell lines and infection conditions. U2OS and HeLa cells were seeded at a density of 25,000 cells per well in a 12-well tissue culture plate and allowed to adhere overnight. The next day, cells were infected with the indicated viruses for 24 h, and then treated overnight with either vehicle, 1μM doxorubicin, or 50 μM etoposide. 48 hours post infection, cells were processed for comet assay using the OxiSelect Comet Assay kit (Cell Biolabs #STA-351) and following the manufacture’s alkaline comet assay protocol. Briefly, cells were harvested, washed once, and resuspended in PBS. Cells were then mixed with OxiSelect comet agarose (1:10 cells to agarose ratio) and solidified on an OxiSelect Comet slide. Slides were then incubated in lysis buffer followed by incubation in alkaline solution. Gel electrophoresis was performed by transferring the slide to an electrophoresis apparatus filled with alkaline electrophoresis solution. Slides were then subjected to 20 volts for 25 min at constant 300 mA. Following electrophoresis, slides were washed with distilled water and 70% ethanol and then stained with 1X Vista Green DNA Dye for 15 min at room temperature. Comets were imaged on an EVOS M500 fluorescence microscope using a 20x objective and data were analyzed using the OpenComet plugin for ImageJ. All experiments were repeated at least 3 independent times between two investigators.

### Immunoblotting analysis

For immunoblotting assays, HEK293T cells were plated at a seeding density of approximately 300,000 cells per well in a 12-well tissue culture plate. The next day, cells were transfected with the indicated viruses for 48 h prior to collection and pelleting. Cell pellets were resuspended in 200 μL RIPA (50mM Tris [pH 8.0], 1mM β-mercaptoethanol, 150 mM NaCl, 1% Triton X-100, 0.5% deoxycholate, 0.1% SDS) with a protease and phosphatase inhibitor cocktail (Thermo Scientific #78440). 10% of the total cell lysate was combined with 5X loading dye (62.5 mM Tris [pH 6.8], 20% glycerol, 2% SDS, 5% β-mercaptoethanol, 0.05% bromophenol blue). Samples were separated by a 12% SDS-PAGE gel and transferred to 0.2 μm PVDF membrane (Thermo Scientific #78440). Membranes were blocked in 5% milk in PBS for 1 h and then incubated with primary antibody, αGAPDH (1:1000, sc-32233), αTubulin (1:1000, Sigma T5168), αHA (1:5000, CST C29F4) and αVif (1:1000, NIH Reagent Program #319) in blocking solution overnight at 4°C. The next day, blots were washed four times with PBST (0.05% Triton X-100 in PBS) and incubated with secondary antibody, αmouse HRP (1:4000, sc-525409) diluted in 5% milk in PBS for 1h. Blots were incubated with West Pico PLUS chemiluminescent substrate (Thermo Scientific #34580) for 5 min before visualization on GE ImageQuant LAS 500 imager.

### Flow cytometry

All flow cytometry experiments were repeated three independent times and representative histograms are depicted from one experiment. For degradation assays in **Fig 1**, stable HEK293T cell lines expressing APOBEC3G-eGFP or eGFP-B56A were seeded at 150,000 cells per well in a 12-well. The following day, cells were transfected with 600 ng of Vif constructs using PEI. At 48 h post-transfection, cells were harvested using PBS/EDTA, centrifuged at 500 x g for 10 min, then resuspended in 2% FBS in PBS. Samples were subjected to flow cytometry and quantification of fluorescence intensity was performed using a Becton Dickinson FACScan flow cytometer.

For patient-derived isolates and HIV-1 subtype consensus sequences, HEK293T cell lines were seeded at a density of 150,000 cells per well in a 12-well plate. The following day, cells were transfected with 300 ng of the indicated eGFP-B56 constructs and 300 ng of the indicated Vif isolate using PEI and allowed to incubate for 48 h. At 48 hours post-transfection, cells were processed for flow cytometry following procedure describe above. Quantification of fluorescence intensity was performed using an Invitrogen Attune NxT flow cytometer.

### Drugs and chemical inhibitors

To induce DDR signaling responses depicted in **Figs 2** and **3**, indicated cell lines were infected for 48 h then incubated with 50 μM etoposide (Fisher, #AAJ63651MC) for 30 min at 37°C, and then processed for immunofluorescence microscopy. To specifically induce ATR and downstream signaling responses, cells were infected for 48 h then incubated with 50 μM camptothecin (Fisher, #AAJ6252303) for 1 h at 37°C prior to immunofluorescence microscopy analysis. For ATM and ATR inhibitor experiments, 24 hours post infection cells were incubated with either 10 nM ATM inhibitor (Fisher, #AZD1390), 10 μM ATR inhibitor (Fisher, #NU6027) or 3 mM caffeine for 24 h. The DNA-PK inhibitor was used a 10 μm for 24 hours. Cells were then processed for immunofluorescence microscopy for γH2AX focus formation. For detection of TRIM24 and pTRIM28 in the presence and absence of inhibitor treatments, the same workflow described above was repeated with the exception that cells were treated with 50 μM etoposide for 4 h and 30 min, respectively, before immunostaining. Bleomycin was used at 5 μg/ml for 30 mins and Mitomycin C (MMC) was used at 2 μg/ml for 8 hours.

### Quantification and statistical analysis

All flow cytometry data were analyzed using FlowJo v10 software. The data shown are representative results from one of three independent experiments. Associated histogram profiles of flow cytometry data in **Fig 1** were generated using FlowJo. Focus formation and mean fluorescence intensity (MFI) analyses was conducted using ImageJ software and analyzed using GraphPad Prism 6 software. Briefly, boundaries of infected cell nuclei were defined using DAPI staining as an indicator and then foci were quantified using the “find maxima” feature and eGFP mean fluorescence intensity was defined by analyzing integrated pixel intensity of the defined nuclear area minus the background signal intensity of an adjacent area with identical dimensions. The B56 protein structure depicted in **Fig 1** was generated using Chimera protein modeling software (PDB #2JAK). Statistical analyses were performed using either an unpaired two-tailed Student’s *t-*test or a one-way ANOVA analysis in GraphPad Prism 8 after confirming that all data followed a normal distribution. DDR signaling schematics were generated using BioRender.

## Supporting information

S1 Data Excel spreadsheet containing, in separate sheets, underlying numerical data used to generate the indicated figure panels. (XLSX)

S1 Fig Flow cytometry analysis and DDR inhibition in Vif_SLQ-AAA_ expressing cells. (**A**) Gating strategy for flow cytometry experiments assessing GFP-tagged substrate depletion. Events were stratified by forward- (FSC) and side-scatter (SSC), then by mCherry fluorescence intensity, and then assessed for eGFP fluorescence. (**B**) Bar graph representation of 3 independent flow cytometry experiments of transfected cells with the indicated Vif expression constructs against the indicated substrates. (**C**) Quantification and representative images for COMET assays of U2OS cells infected with the indicated viruses treated with DMSO or the indicated concentration of etoposide (n = 50). (**D**) Left, representative images of U2OS cells infected with the indicated viruses, treated with etoposide for 30 minutes, and then stained for gH2AX. Right, quantification of gH2AX staining intensity from one representative experiments (n = 50). Scale bars = 10 μm.
(TIF)

S2 Fig Phosphorylated and total protein for the indicated DNA repair proteins treated with etoposide. (**A-E**) Representative images of the indicated total or phosphorylated DNA repair proteins (pDNA-PK, pSer 2056; gH2AX, pSer 139). U2OS cells were infected with the indicated viruses for 48 hours prior to etoposide treatment and immunofluorescence microscopy analysis. (**F**) Left, same as above, with the exception that the indicated cell types were infected with wild-type (WT) LxxIxE or mutant AxxAxA (Ala) B56-peptide inhibitors. Right, quantification of the indicated cell type infected with the indicated peptide viruses (n = 50). Scale bars = 10 μm.
(TIF)

S3 Fig Expression analysis and flow cytometry histograms of patient-derived Vif isolates. (**A**) Immunoblot analyses of patient-derived Vif isolates transiently expressed in HEK293T cells. Because the Vif antibody is monoclonal, it fails to detect several of the patient-derived isolates. To overcome this issue, patient isolates that were not recognized by the native antibody were N-terminally tagged with an HA-epitope to assess expression. (**B**) Flow cytometry histograms from selected patient isolates characterized for DDR responses in **Fig 3B**. The gating strategy follows the same one depicted in S1 Fig, with the eGFP-tagged substrate indicated below each dataset.
(TIF)

S4 Fig Phosphorylated DNA repair proteins treated with camptothecin or etoposide. (**A**-**C**) Representative images of the indicated phosphorylated DNA repair proteins from infected U2OS cells treated with camptothecin (pATR, pSer 428; pCHK1, pSer 345; gH2AX, pSer 139). U2OS cells were infected with the indicated viruses for 48 hours prior to camptothecin treatment and immunofluorescence microscopy analysis. (**D**) Left, Representative images of U2OS cells infected with the indicated viruses, treated with etoposide for 30 minutes, and then stained for pATR. Right, quantification of pATR staining intensity from one representative experiment (n = 50). Scale bars = 10 μm.
(TIF)

S5 Fig Phosphorylated and total protein for the indicated DNA repair proteins treated with etoposide. (**A**-**G**) Representative images of the indicated phosphorylated DNA repair proteins (pATM, pSer 1981; pNBS1, pSer 343; pMRE11, pSer 678; pCHK2, pThr 68). U2OS cells were infected with the indicated viruses for 48 hours prior to etoposide treatment and immunofluorescence microscopy analysis. (**H**) Left, Representative images of HeLa cells infected with the indicated viruses and then stained for pCHK2 48-hours post infection. Right, quantification of pCHK2 staining intensity from one representative experiment (n = 50). Scale bars = 10 μm.
(TIF)

S1 Table Statistical analyses of infected cells following inhibitor treatments. (TIF)
