# Supplementary figures and images for: Inhibition of ATM-directed antiviral responses by HIV-1 Vif
Source: PLoS Pathog. 2023 Sep 5;19(9):e1011634. doi: 10.1371/journal.ppat.1011634 (PMC10503699; doi:10.1371/journal.ppat.1011634)

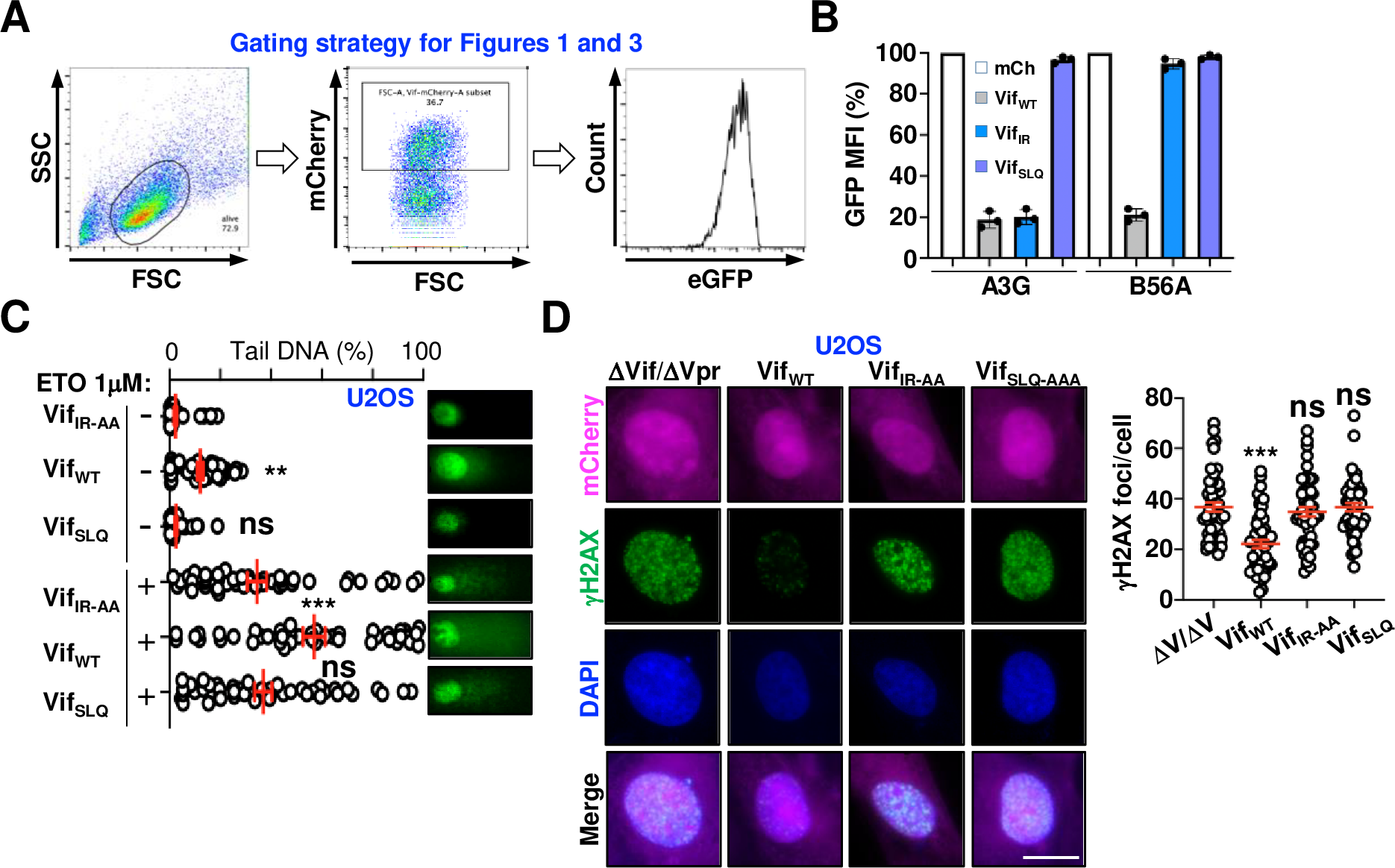

Supplement: S1 Fig — (A) Gating strategy for flow cytometry experiments assessing GFP-tagged substrate depletion. Events were stratified by forward- (FSC) and side-scatter (SSC), then by mCherry fluorescence intensity, and then assessed for eGFP fluorescence. (B) Bar graph representation of 3 independent flow cytometry experiments of transfected cells with the indicated Vif expression constructs against the indicated substrates. (C) Quantification and representative images for COMET assays of U2OS cells infected with the indicated viruses treated with DMSO or the indicated concentration of etoposide (n = 50). (D) Left, representative images of U2OS cells infected with the indicated viruses, treated with etoposide for 30 minutes, and then stained for gH2AX. Right, quantification of gH2AX staining intensity from one representative experiments (n = 50). Scale bars = 10 μm. (TIF) [file ppat.1011634.s002.tif]

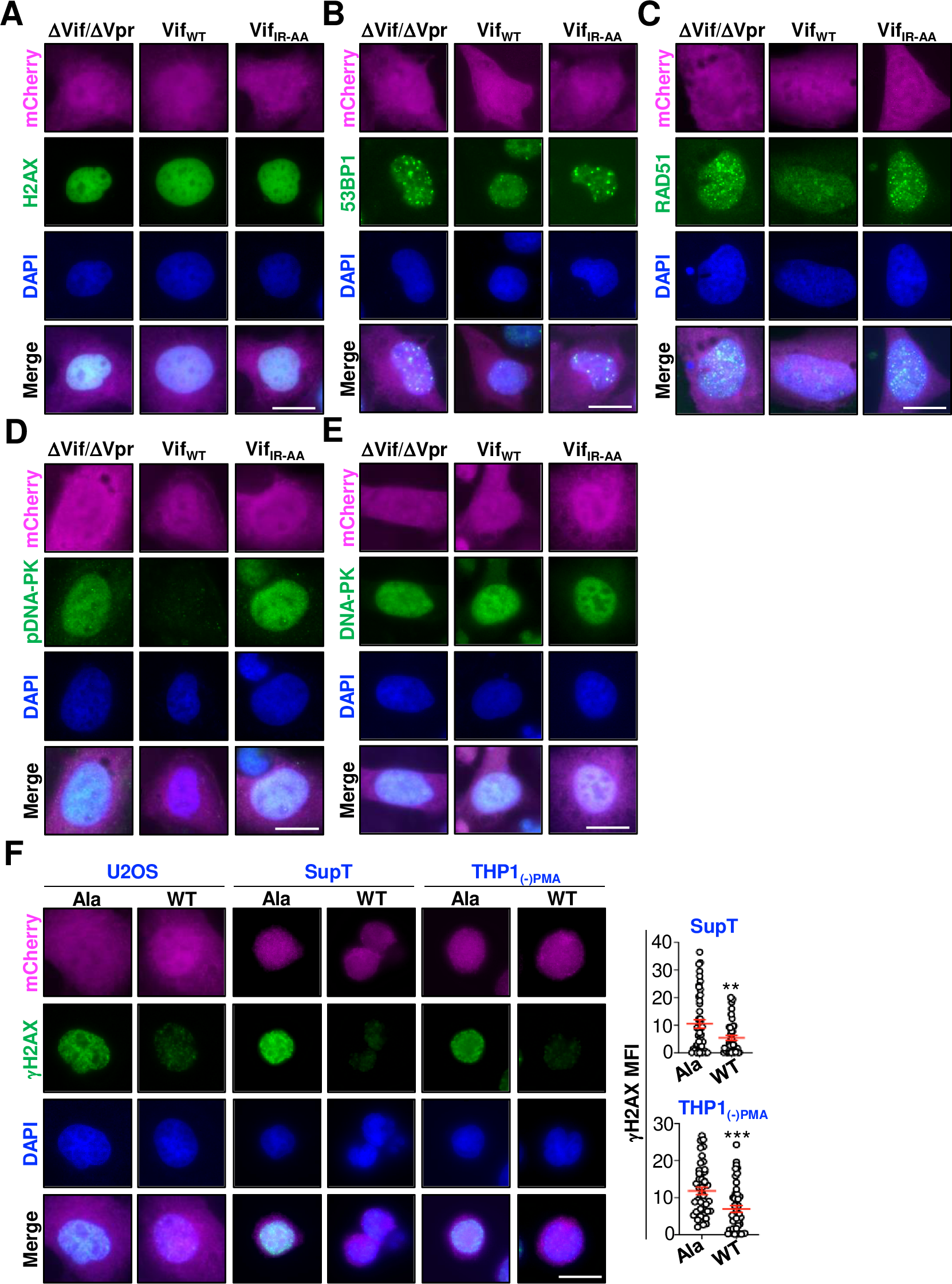

Supplement: S2 Fig — (A-E) Representative images of the indicated total or phosphorylated DNA repair proteins (pDNA-PK, pSer 2056; gH2AX, pSer 139). U2OS cells were infected with the indicated viruses for 48 hours prior to etoposide treatment and immunofluorescence microscopy analysis. (F) Left, same as above, with the exception that the indicated cell types were infected with wild-type (WT) LxxIxE or mutant AxxAxA (Ala) B56-peptide inhibitors. Right, quantification of the indicated cell type infected with the indicated peptide viruses (n = 50). Scale bars = 10 μm. (TIF) [file ppat.1011634.s003.tif]

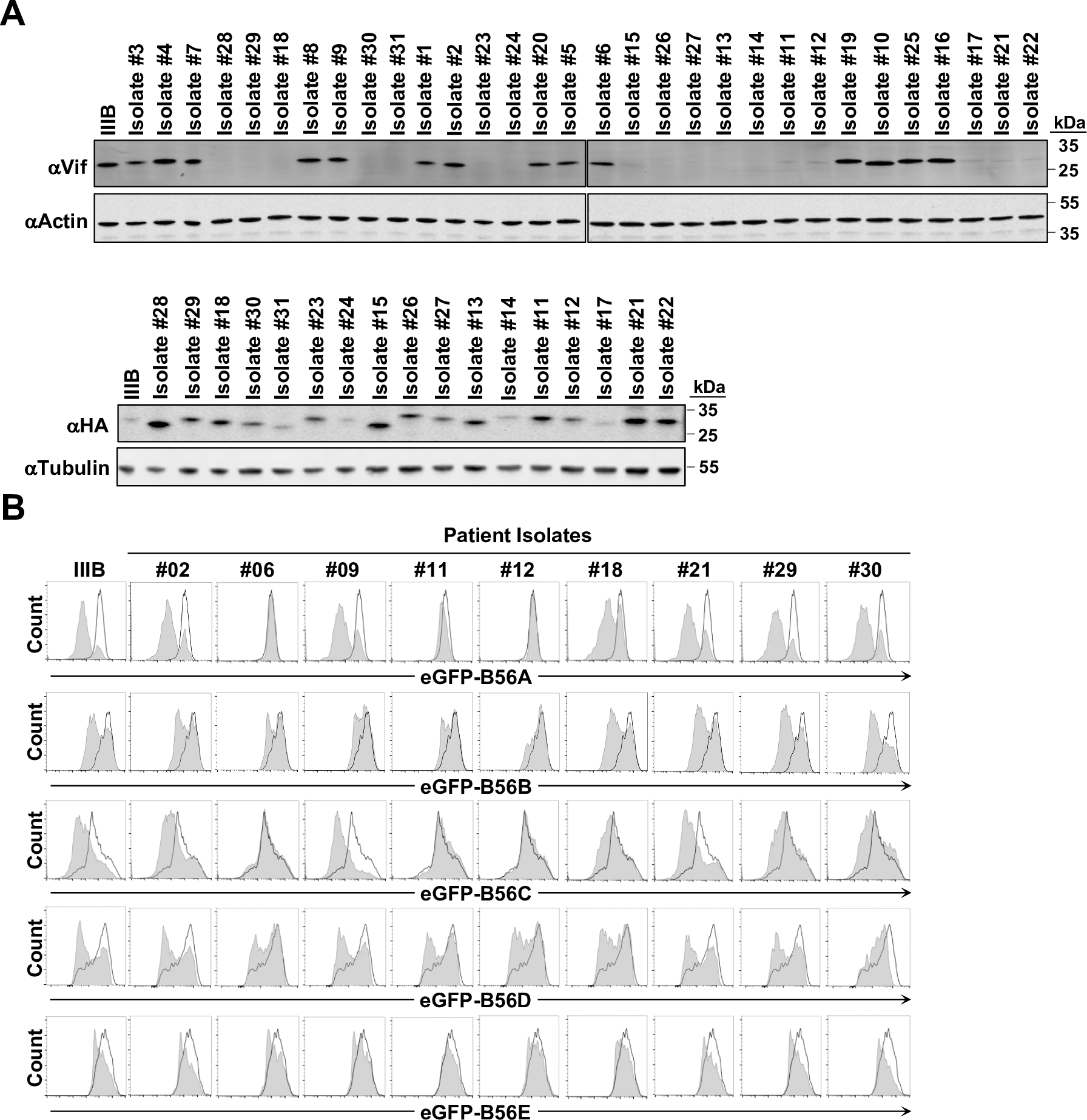

Supplement: S3 Fig — (A) Immunoblot analyses of patient-derived Vif isolates transiently expressed in HEK293T cells. Because the Vif antibody is monoclonal, it fails to detect several of the patient-derived isolates. To overcome this issue, patient isolates that were not recognized by the native antibody were N-terminally tagged with an HA-epitope to assess expression. (B) Flow cytometry histograms from selected patient isolates characterized for DDR responses in Fig 3B. The gating strategy follows the same one depicted in S1 Fig, with the eGFP-tagged substrate indicated below each dataset. (TIF) [file ppat.1011634.s004.tif]

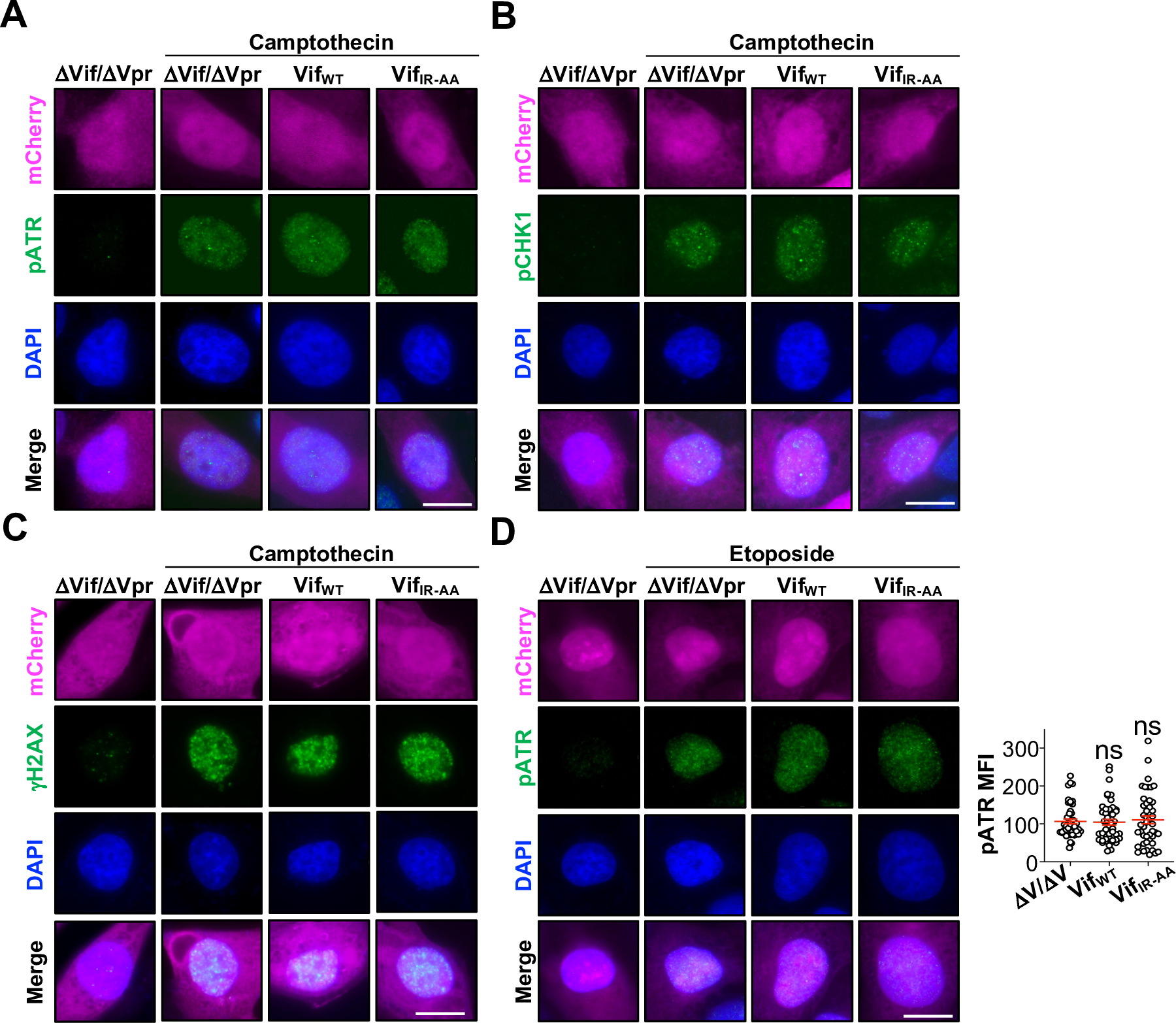

Supplement: S4 Fig — (A-C) Representative images of the indicated phosphorylated DNA repair proteins from infected U2OS cells treated with camptothecin (pATR, pSer 428; pCHK1, pSer 345; gH2AX, pSer 139). U2OS cells were infected with the indicated viruses for 48 hours prior to camptothecin treatment and immunofluorescence microscopy analysis. (D) Left, Representative images of U2OS cells infected with the indicated viruses, treated with etoposide for 30 minutes, and then stained for pATR. Right, quantification of pATR staining intensity from one representative experiment (n = 50). Scale bars = 10 μm. (TIF) [file ppat.1011634.s005.tif]

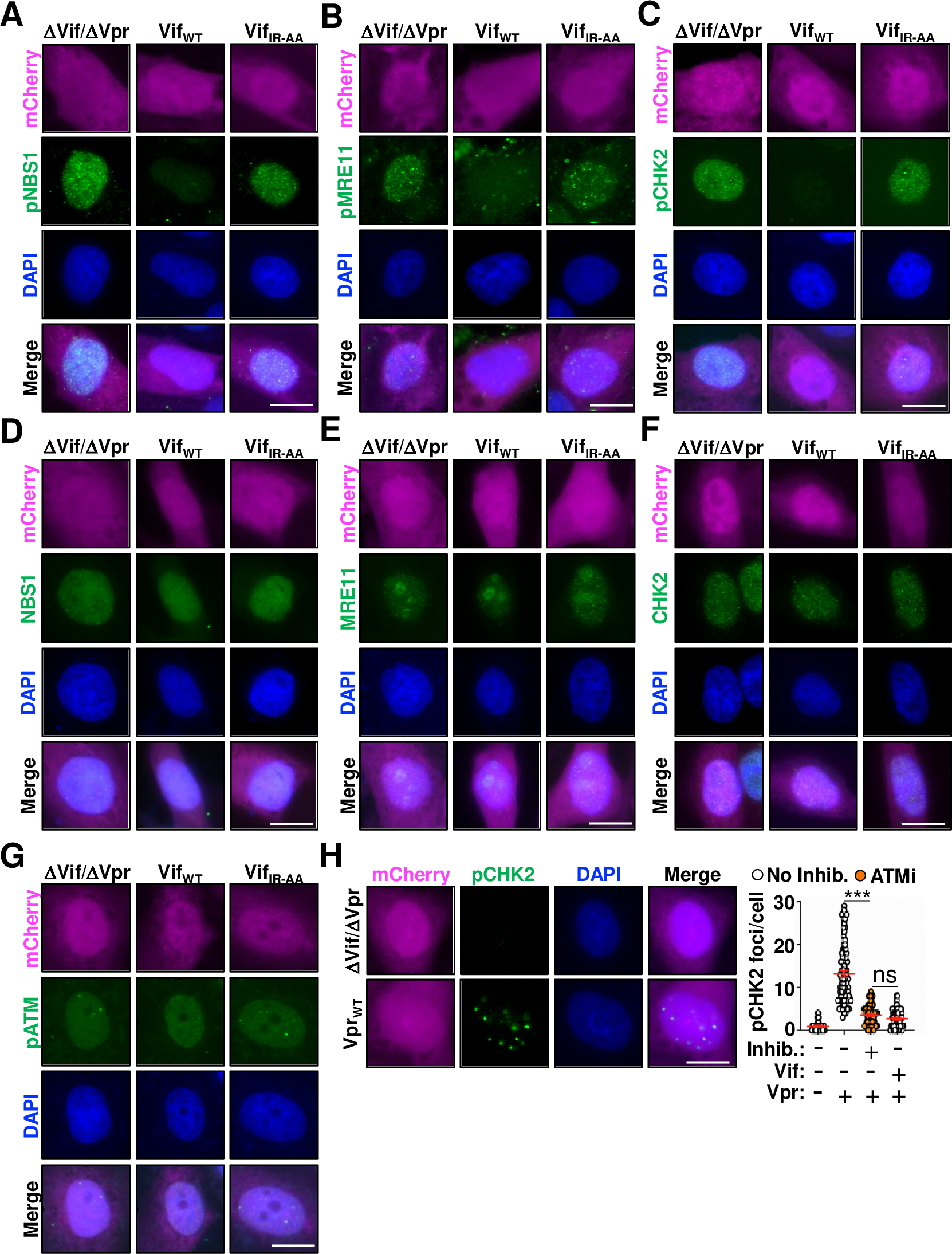

Supplement: S5 Fig — (A-G) Representative images of the indicated phosphorylated DNA repair proteins (pATM, pSer 1981; pNBS1, pSer 343; pMRE11, pSer 678; pCHK2, pThr 68). U2OS cells were infected with the indicated viruses for 48 hours prior to etoposide treatment and immunofluorescence microscopy analysis. (H) Left, Representative images of HeLa cells infected with the indicated viruses and then stained for pCHK2 48-hours post infection. Right, quantification of pCHK2 staining intensity from one representative experiment (n = 50). Scale bars = 10 μm. (TIF) [file ppat.1011634.s006.tif]

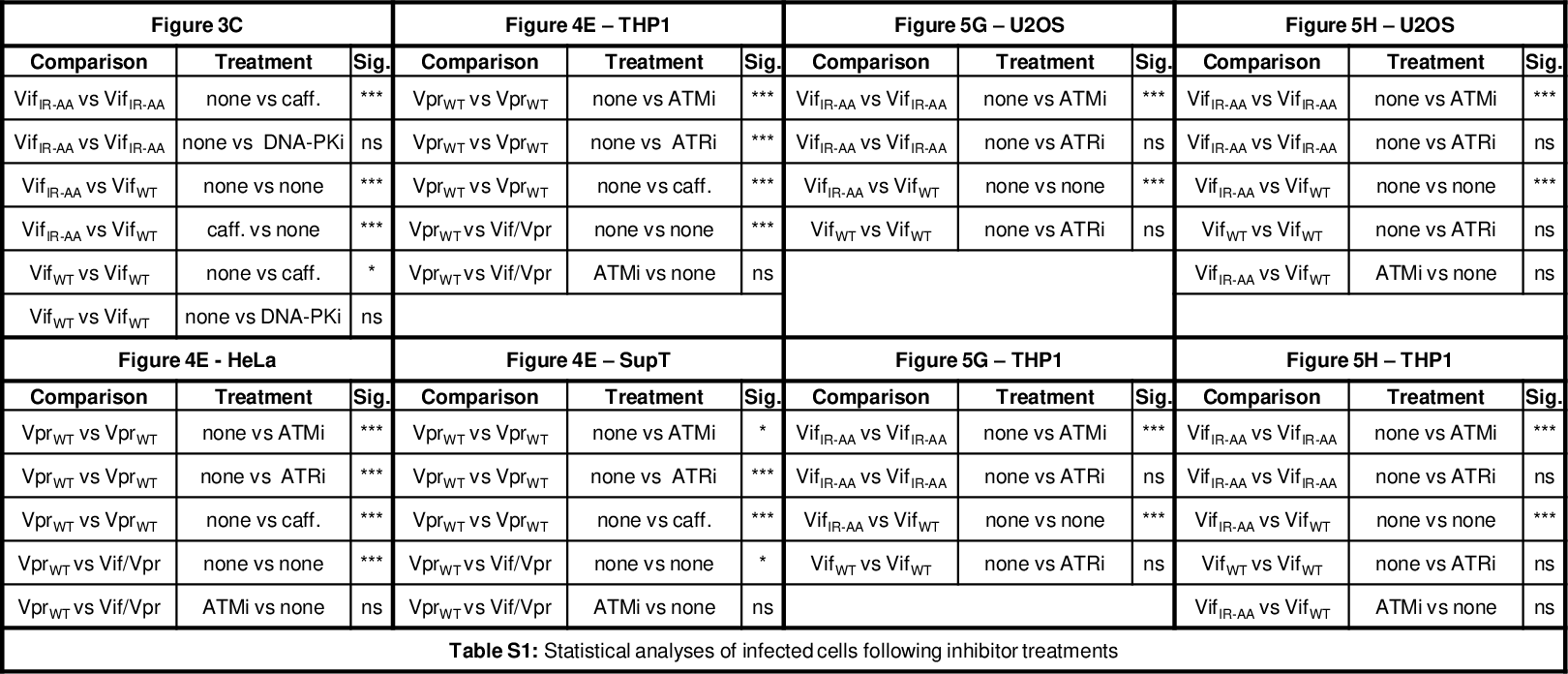

Supplement: S1 Table — (TIF) [file ppat.1011634.s007.tif]
